# Serotype distribution, clinical characteristics, and antimicrobial resistance of pediatric invasive pneumococcal disease in Colombia during PCV10 mass vaccination (2017–2022)

**DOI:** 10.3389/fmed.2024.1380125

**Published:** 2024-05-22

**Authors:** Germán Camacho-Moreno, Aura Lucia Leal, Jaime Patiño-Niño, Pablo Vasquez-Hoyos, Ivan Gutiérrez, Sandra Beltrán, Martha I. Álvarez-Olmos, Ana-Cristina Mariño, Juan Pablo Londoño-Ruiz, Rocio Barrero, Juan Pablo Rojas, Fabio Espinosa, Catalina Arango-Ferreira, María Alejandra Suarez, Monica Trujillo, Eduardo López-Medina, Pio López, Wilfrido Coronell, Nicolas Ramos, Alejandro Restrepo, Anita Montañez, Vivian Marcela Moreno

**Affiliations:** ^1^Red Neumocolombia, Bogotá, Colombia; ^2^Universidad Nacional de Colombia, Bogotá, Colombia; ^3^HOMI, Fundación Hospital Pediatrico de la Misericordia, Bogotá, Colombia; ^4^Hospital Infantil Universitario de San José, Bogotá, Colombia; ^5^Grupo Para el Control de la Resistencia Bacteriana en Bogotá, GREBO, Bogotá, Colombia; ^6^Fundación Valle del Lili, Cali, Colombia; ^7^Clínica Infantil Colsubsidio, Bogotá, Colombia; ^8^Clinicas Colsanitas—Clinica Santa Maria del Lago, Bogotá, Colombia; ^9^Clínicas Colsanitas—Clínica Reina Sofia pediátrica y Mujer, Bogotá, Colombia; ^10^Fundación Cardioinfantil—Instituto de Cardiología, Bogotá, Colombia; ^11^Hospital Militar Central, Bogotá, Colombia; ^12^Hospital Universitario Clínica San Rafael, Bogotá, Colombia; ^13^Unidad de Servicios de Salud Santa Clara, Subred Centro Oriente, Bogotá, Colombia; ^14^Fundación Clínica Infantil Club Noel, Cali, Colombia; ^15^Universidad Libre Seccional Cali, Cali, Colombia; ^16^Universidad del Valle, Cali, Colombia; ^17^Hospital Universitario San Vicente Fundación, Medellín, Colombia; ^18^Unidad de Servicio de Salud Tunal, Bogotá, Colombia; ^19^Hospital Universitario San Ignacio, Bogotá, Colombia; ^20^Hospital Pablo Tobón Uribe, Medellín, Colombia; ^21^Centro Médico Imbanaco, Cali, Colombia; ^22^Hospital Universitario del Valle, Cali, Colombia; ^23^Hospital Infantil Napoleón Franco Pareja, Cartagena, Colombia; ^24^Clínica el Bosque—Los Cobos Medical Center, Bogotá, Colombia

**Keywords:** *Streptococcus pneumoniae*, children, serotype, vaccines, antimicrobial resistance, clinical epidemiology

## Abstract

**Introduction:**

Invasive Pneumococcal Disease (IPD) causes significant morbidity and mortality in children under 5 y. Colombia introduced PCV10 vaccination in 2012, and the Neumocolombia network has been monitoring IPD in pediatric patients since 2008.

**Materials and methods:**

This study is a secondary analysis of a prospective cohort involving pediatric patients with IPD admitted to 17 hospitals in Colombia, from January 1st, 2017, to December 31st, 2022. We present data on serotypes (Spn), clinical characteristics, and resistance patterns.

**Results:**

We report 530 patients, 215 (40.5%) were younger than 24 months. Among these, 344 cases (64.7%) presented with pneumonia, 95 (17.9%) with primary bacteremia, 53 (10%) with meningitis, 6 (1.1%) had pneumonia and meningitis, and 32 (6%) had other IPD diagnosis. The median hospital stay was 12 days (RIQ 8–14 days), and 268 (50.6%) were admitted to the ICU, of whom 60 (11.3%) died. Serotyping was performed in 298 (56.1%). The most frequent serotypes were Spn19A (51.3%), Spn6C (7.7%), Spn3 (6.7%), Spn6A (3.6%), and Spn14 (3.6%). Of 495 (93%) isolates with known susceptibility, 46 (9.2%) were meningeal (M) and 449 (90.7%) non-meningeal (NM). Among M isolates, 41.3% showed resistance to penicillin, and 21.7% decreased susceptibility to ceftriaxone. For NM isolates, 28.2% had decreased susceptibility to penicilin, and 24.2% decreased susceptibility to ceftriaxone. Spn19A showed the highest resistant to penicillin at 47% and was linked to multiresistance.

**Conclusion:**

The prevalence of PCV10-included serotypes decreased, while serotypes 19A and 6C increased, with Spn19A being associated with multiresistance. These findings had played a crucial role in the decision made by Colombia to modify its immunization schedule by switching to PCV13 in July 2022.

## Introduction

*Streptococcus pneumoniae* infections are of great concern due to the high rates of morbidity and mortality, especially in children under 5 years of age (1, 2). Pneumococcus colonizes 42% of these children (2), predisposing them to noninvasive diseases such as otitis media, acute sinusitis, and pneumonia, as well as invasive pneumococcal disease (IPD), such as bacteremic pneumonia, meningitis, primary bacteremia, peritonitis, and arthritis among others (1, 2). More than 100 pneumococcal serotypes have been identified, each of which presents variations in frequency, invasiveness, and resistance to antibiotics (3, 4). Although penicillin and cephalosporins have been the treatments of choice, sensitivity to these beta-lactams has decreased due to the emergence of multiresistant clones (5–7).

Pneumococcal conjugate vaccines (PCVs) have achieved a notable reduction in disease burden and have the potential to decrease resistance to antibiotics, as long as they cover serotypes with a high proportion of resistance (1, 8–11). However, the impact of these PCVs is influenced by multiple factors, such as immunogenicity, efficacy/effectiveness against disease and nasopharyngeal carriage, vaccination coverage rates, vaccination schedule, quality of epidemiological surveillance, and serotype replacement (4, 12, 13). This last phenomenon has been observed with the introduction of PCV7 and is characterized by the emergence of serotypes not covered by the vaccine that occupy the ecological niche that leaves a decrease in vaccine serotypes, which led to the introduction of PCVs with additional valences, such as PCV10 currently administered in 24 countries and PCV13 administered in 121 countries (4, 13–16). Vaccines with more valences, such as PCV15 and PCV20, have recently been developed (17).

In Colombia, vaccination against pneumococcus began in 2006 with PCV7, initially targeting children under 2 years of age at high risk of IPD. Since 2008, the vaccine was administered universally initially in Bogotá, and since 2009 it was extended to the departments of Colombia with the highest mortality from acute respiratory infection. In the 2010–2011 period, given the withdrawal of PCV7 from the market, PCV13 was administered in Bogotá and in the higher risk departments. Based on a cost-effectiveness study (18), since January 2012, PCV10 has been administered universally, using a 2 + 1 scheme administered at 2 months, 4 months, and 12 months of age, with vaccination coverage between 85 and 89%. In July 2022, based on epidemiological data and cost-effectiveness studies (19, 20). Colombia switched to PCV13 for the cohort of children born on or after May 1, 2022, using the same 2 + 1 scheme. A laboratory-based surveillance system is available for the distribution of serotypes and resistance in Colombia, in which the isolates of meningitis must be sent to departmental and district health secretaries and from there to the National Institute of Health (INS), which performs serotyping. The shipment of other invasive isolates is voluntary, so clinical information on these isolates is scarce (21, 22).

This study details the clinical characteristics, distribution of serotypes, and resistance to antibiotics in a cohort of children treated during the mature stage of vaccination with PCV10, at 17 hospitals in Colombia. These hospitals are part of the Neumocolombia network, which monitors the behavior of the IPD in Colombia since 2008.

## Materials and methods

### Description of the cohort

This was a secondary analysis of a prospective cohort composed of pediatric patients with IPD. The patients were admitted to 17 hospitals in Colombia, distributed as follows: Ten in Bogotá, four in Cali, two in Medellín, and one in Cartagena, between January 1, 2017 and December 31, 2022. Sampling was carried out consecutively, focusing on those with a positive culture or positive molecular test of a normally sterile site, and each patient was followed up throughout the hospitalization.

### Case definition

An IPD was defined by the isolation of *Streptococcus pneumoniae* in culture or by molecular testing of a normally sterile site, such as blood, cerebrospinal fluid (CSF), pleural fluid (PF), synovial fluid (SF), peritoneal fluid (PeF), or bone (B). It is considered that a patient has IPD if pneumococcus is documented by either of the two methods (culture or molecular test) or by both. This definition was applied regardless of the signs and symptoms present in the patient. To identify the patients, the Whonet 5.5^®^ system (World Health Organization) was used in each of the participating hospitals during the study period (23). Clinical information was collected through the medical records of the patients using a specific format developed in Microsoft Excel^®^. The data collected included sociodemographic variables, clinical and laboratory findings, and outcomes.

### Laboratory methods

The isolates were identified through culture or molecular testing. For isolates obtained from sterile sites, identification and susceptibility testing were conducted using automated methods. Sixteen centers utilized Vitek 2^™^ (Biomérieux, L’Étoile, France), while one center employed BD Phoenix^™^ (Becton Dickinson). CLSI 2022 criteria were applied to determine antibiotic susceptibility breakpoints (24).

Isolates detected solely through molecular testing (BioFire^®^ Meningoencephalitis Panel, BioFire^®^ pneumonia panel, BioFire^®^ Blood Culture Identification 2 (BCID2) Panel) did not undergo antibiotic susceptibility testing.

### Serotyping

As part of routine surveillance, the culture isolates and cerebrospinal fluid (CSF) positive by polymerase chain reaction PCR, were sent to departmental or district health secretaries, where the isolates were reconfirmed and then sent to the Microbiology Group—National Reference Laboratory for Instituto Nacional de Salud (INS), the culture isolates were serotyped by the Quellung method and agglutination using commercial antisera (DIFCO, Becton Dickinson^®^) which allows for the identification of 88 serotypes, and by PCR in cases where the antiserum result reports serogroup 16, or in cerebrospinal fluid samples where the isolation was detected solely by PCR. The serotype data were taken from the report issued by the INS (21, 22, 25, 26).

### Statistical analysis

Descriptive statistical techniques were used to analyze the data. Measures of central tendency and dispersion were calculated for continuous variables such as age and length of hospital stay. Proportions are given for categorical variables, including disease types and clinical outcomes. Subgroups of vaccination status and identified pneumococcal serotype were examined. Antibiotic resistance patterns were analyzed by susceptibility subtypes of different serotypes.

### Ethical aspects

The research protocol was initially approved by the Ethics and Research Committee of HOMI, Hospital pediátrico la Misericordia, through Resolution No. 015 CEI 5018, and subsequently approved by the ethics and research committees of each participating institution. In hospitals where the research committee requested it (such as Hospital Infantil Universitario de San José y el Hospital Universitario Clínica San Rafael), informed consent was obtained from the parents. In other hospitals, the ethics and research committee waived the need for informed consent, considering it to be a low-risk study.

## Results

The study included 530 patients, with an annual average of 88 patients. Of these, 290 cases were recorded between 2017 and 2019, and 240 cases were recorded between 2020 and 2022. A decrease in the number of isolates was observed in 2020 (*n* = 36) and 2021 (*n* = 68), with a further increase occurring in 2022 (*n* = 137).

The median age was 29 months (IQR 12–50). A total of 215 patients (40.5%) were younger than 24 months, 205 (38.7%) were between 24 and 59 months, and 110 (20.7%) were between 60 and 214 months. Regarding the clinical manifestations, 344 patients (64.9%) presented with pneumonia, 134 with pleural effusion (38.9%), 95 (27.6%) with pneumonia complicated with empyema, and 58 (16.8%) with necrotizing pneumonia. In 130 of the 134 patients with pneumonia with effusion (Supplementary Table S1). There were 95 (17.9%) patients with primary bacteremia. There were 53 (10%) patients with meningitis. Thirty-two (6%) other diagnoses were logged: 12 patients with osteoarticular infection were identified, one of them, identified only by molecular testing of joint fluid. While culture identified six cases of mastoiditis, six cases of skin and soft tissue infection, five cases of primary peritonitis, two cases of catheter-associated bacteremia, and one case of subdural empyema secondary to pansinusitis. The clinical characteristics and outcomes are shown in Table 1.

**Table 1:** Patient characteristics, by age, diagnosis, and outcomes.

Clinical presentation	Variable	<24 m	24–59 m	>60 m	Total
Pneumonía	Median hospital stay. Med (IQR)	14 (7–23)	14 (7–23)	10 (5–17)	13 (7–21)
	Admission to PICU *n* (%)	79 (56.4)	81 (51.9)	27 (56.3)	187
	Median PICU days. Med (IQR)	9 (5–16)	8 (3–14)	5 (3–11)	8 (4–14)
	Lethality *n* (%)	15 (10.7)	10 (6.4)	5 (10.4)	30 (8.7)
	Total patients *n* (%)	140	156	48	344 (64.9)
Primary bacteremia	Median hospital stay. Med (IQR)	8 (1–15)	7 (6–13)	15 (10–31)	11 (5–16)
	Admission to PICU *n* (%)	18 (45)	7 (23.3)	9 (36)	34 (35.8)
	Median PICU days. Med (IQR)	4 (1–10)	6 (3–9)	13 (8–17)	7 (3–13)
	Lethality *n* (%)	9 (22.5)	0 (0)	4 (16)	13 (13.7)
	Total patients *n* (%)	40	30	25	95 (17.9)
Meningitis	Median hospital stay. Med (IQR)	12 (3–19)	18 (15–23)	14 (9–15)	14 (7–19)
	Admission to PICU *n* (%)	13 (68.4)	6 (75)	16 (61.5)	35 (66%)
	Median PICU days. Med (IQR)	10 (3–12)	10 (2–16)	3 (2–6)	6 (3–11)
	Lethality *n* (%)	8 (42.1)	1 (12.5)	5 (19.2)	14 (26.4)
	Total patients *n* (%)	19	8	26	53 (10)
Pneumonía and meningitis	Median hospital stay. Med (IQR)	17 (8–20)	0 (0)	19	17 (8–20)
	Admission to PICU *n* (%)	5 (100)	0 (0)	1 (100)	6 (100)
	Median PICU days. Med (IQR)	11 (5–12)	0 (0)	19	18 (8–20)
	Lethality *n* (%)	2 (40%)	0 (0)	1 (100)	3 (50)
	Total patients *n* (%)	5	0	1	6 (1,1)
Other diagnoses	Median hospital stay. Med (IQR)	16 (11–37)	11 (7–14)	12 (7–19)	12 (8–19)
	Admission to PICU *n* (%)	1 (9,1)	1 (9,1)	4 (40)	6 (18,7)
	Median PICU days. Med (IQR)	3	7	5 (4–17)	5 (4–7)
	Lethality *n* (%)	0 (0)	0 (0)	0 (0)	0 (0)
	Total pacientes *n* (%)	11	11	10	32 (6)
All diagnoses	Median hospital stay. Med (IQR)	13 (7–22)	12 (7–18)	13 (7–19)	12 (8–14)
	Admission to PICU *n* (%)	116 (53.9)	95 (46.3)	57 (51.8)	268 (50.6)
	Median PICU days. Med (IQR)	9 (4–14)	7 (4–14)	6 (3–11)	9 (6–13)
	Lethality *n* (%)	34 (15.8)	11 (5.4)	15 (13.6)	60 (11.3)
	Total patients *n* (%)	215	205	110	530 (100)

PICU, Pediatric Intensive Care Unit; Med (IQR), Median (Interquartile Range); n (%), Number of patients and percentage of the total. Pneumonia and Meningitis: Patients diagnosed with both conditions. Other diagnoses *: included osteoarticular infections, mastoiditis, skin and soft tissue infections, primary peritonitis, catheter-associated bacteremia and subdural empyema secondary to pansinusitis. All: Sum of all patients in the listed categories. The data are presented according to age group. Lethality refers to the percentage of deceased patients within each diagnostic and age group.

A total of 473 patients (89.2%) were diagnosed exclusively through positive cultures (blood culture and/or CSF, pleural fluid, synovial fluid, or peritoneal fluid culture) without undergoing a molecular test. Another 46 patients (8.8%) were diagnosed by positive cultures plus a positive blood or fluid molecular test. Eleven patients (2%) were diagnosed only through a positive molecular test, with negative culture results. Meningitis was the pathology where the greatest impact of the use of the molecular panel was observed, where 7 patients had positive CSF PCR with negative cultures, increasing the diagnosis by 13.2%. Detailed information is presented in Supplementary Table S1.

### Vaccination

Regarding vaccination status, information was obtained for 410 patients (77.3%), 31 (7.6%) of whom did not receive any dose of vaccine, 379 (92.4%) of whom received at least one dose: 367 (89.5%) at least one dose of PCV10, 10 (2.43%) of PCV13, and 2 (0.48%) of PCV7. Specifically, among the 410 patients with known vaccination data, 284 were older than 12 months at the time of diagnosis and were born after the implementation of mass vaccination with PCV 10.Within this subgroup of 284 patients, 5 (1.7%) received no dose, 8 (2,8%) received a single dose, 20 (7%) received two doses, and 251 (88,3%) received three doses. Of the patients who completed the three-dose schedule, 249 were vaccinated with PCV10 and two were vaccinated with PCV13.

### Serotypes

A total of 298 of the 530 isolates (56.2%) were serotyped. A decrease in the serotyping ratio was observed from 61.5% (179/290) in 2017–2019 to 49.5% (119/240) in 2020–2022. The most frequent serotypes were Spn19A (51.3%), Spn6C (7.7%), Spn3 (6.7%), Spn6A (3.6%), and Spn14 (3.6%). Among the identified serotypes, 18 (6%) were included in PCV10, 202 (68%) were included in PCV13 and PCV15, and 212 (71%) were included in PCV20. Isolates of serotypes 22F and 33F were not documented. Considering the cross-protective effects of PCV13, PCV15, and PCV20 against Spn6C, vaccination coverage increased by 7.7% for each vaccine (Supplementary Figure S1).

**Figure 1:**
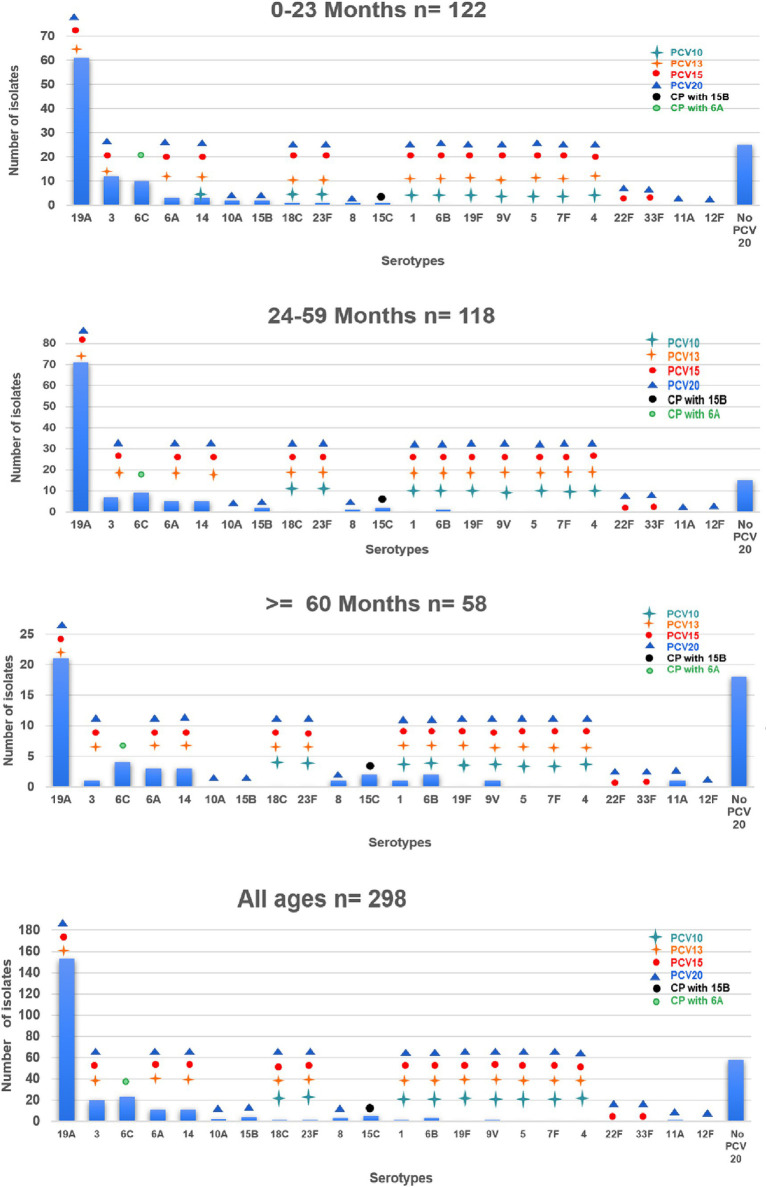
Distribution of *Streptococcus pneumoniae* serotypes by age group and vaccine type. This graph shows the distribution of the different *Streptococcus pneumoniae* serotypes found in the patients according to age group: under 24 months, 24 to 59 months and over 60 months. The following serotypes are classified according to the vaccine: PCV10, PCV13, PCV15, PCV20, and those not included in PCV20. The height of each bar indicates the total number of cases for each serotype within each age group, providing a visual comparison of the prevalence of serotypes in different age cohorts and their coverage by the different vaccines. CP, cross protection.

Regarding the prevalence of serotypes over time, the prevalence of Spn 19A in the period 2017–2019 was 43% (77/179) and in the period 2020–2022, it was 63.8% (76/119). The prevalence of Spn6C in the period 2017–2019 was 5.6% (10/179) and in the period 2020–2022 it was 11.1% (13/119). On the other hand, the prevalence of Spn3 was 9.5% (17/179) in the period 2017–2019 and 2.5% (3/119) in the period 2020–2022 and the prevalence of Spn14 was 5% (9/179) in the period 2017–2019 and 1.7% (2/119) in the period 2020–2022. Figure 1 shows the frequency of serotypes for each age group, identifying the vaccine that includes it.

The serotype was identified in 58.6% (146/249) of the patients who completed the vaccination schedule with PCV10. In this group, the most prevalent serotype was 19A (80 patients, 54.8%), followed by Spn6C (12 patients, 8.6%), Spn3 (11 patients, 7.5%), Spn14 (7 patients, 4.8%), Spn6A (4 patients, 2.7%), and Spn6B (1 patient, 0.7%). Three serotypes included in PCV20 were also identified: three cases of Spn15B, one of Spn8 and one of Spn11A. Twenty-six serotypes found were not included in any of the PCV vaccines.

The mean hospital stay for patients with serotyped isolates was 16.9 days (0–189). Patients with serotype Spn3 had an average stay of 25.6 days (5–58), and those with serotypes not included in PCV20 had a stay of 18.4 days (0–107). The proportion of patients admitted to the PICU was 52.3% for serotyped patients, which was greater for patients with Spn3 (75%). The average stay in the PICU was 11.9 days (1–188), extending to 15 days (1–100) for serotypes not included in PCV20 and to 14.3 days (1–35) for Spn3 (Supplementary Table S2).

### Antimicrobial susceptibility

**Figure 2:**
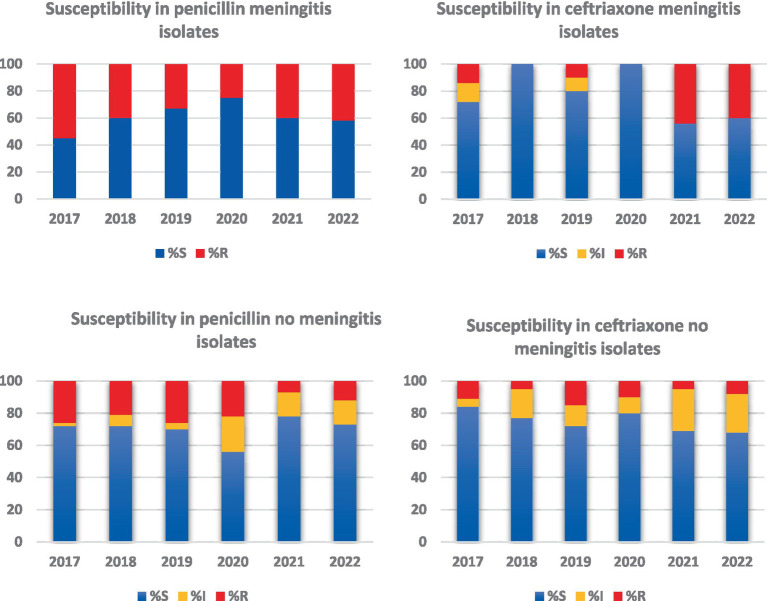
Penicillin and ceftriaxone resistance profiles in meningeal and non-meningeal isolates of *Streptococcus pneumoniae*. This graph compares resistance to penicillin and ceftriaxone in meningeal and non-meningeal isolates of *Streptococcus pneumoniae*. Resistance and decreased sensitivity to these antibiotics are presented separately for each type of isolate. Bars represent the percentage of isolates that showed decreased resistance or sensitivity, providing a clear view of the differences in resistance profiles between meningeal and non-meningeal isolates. This comparison is crucial to understand the dynamics of antibiotic resistance in different clinical presentations of *Streptococcus pneumoniae* infection.

Antibiotic susceptibility profiles were determined for 495 isolates, 93.4% of the total. Of these, 46 (9.2%) were isolated from patients with meningitis (M), and 449 (90.7%) were isolated from patients without meningitis (NM). Among the M isolates, 41.3% were resistant to penicillin, and 21.7% had decreased sensitivity to ceftriaxone (Figure 2). Among the NM isolates, 28.2% had decreased sensitivity to penicillin, and 24.2% had decreased sensitivity to ceftriaxone. Resistance to macrolides was observed in 56% of the patients, resistance to clindamycin in 43.2%, and resistance to trimethoprim-sulfamethoxazole in 51.5%. All the isolates were sensitive to vancomycin, and only 22.5% were sensitive to all the antibiotics.

**Figure 3:**
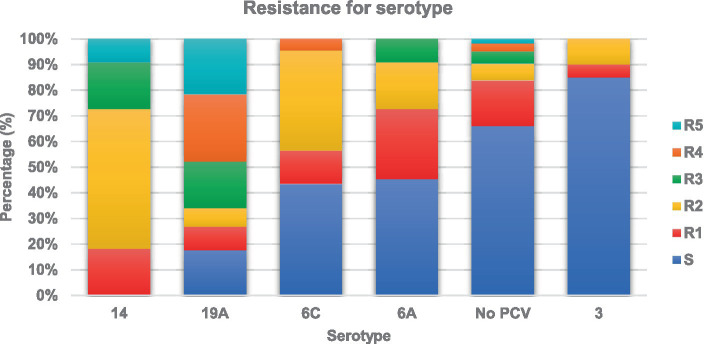
Resistance of *Streptococcus pneumoniae* serotypes to different families of antibiotics. This graph illustrates the resistance of different serotypes of *Streptococcus pneumoniae* to various families of antibiotics. Each bar represents a specific serotype and shows the proportion of resistance to each family of antibiotics, such as penicillins, cephalosporins, macrolides, clindamycin, and trimethoprim-sulfamethoxazole. S is the proportion of isolates susceptible to all antibiotic families, R1 the proportion resistant to 1 family, R2 the proportion resistant to 2 families, R3 the proportion resistant to 3 families, R4 the proportion resistant to 4 families and R5 the proportion of isolates resistant to 5 families of antibiotics. This analysis allows the identification of antibiotic resistance patterns associated with specific serotypes, providing valuable information for the selection of effective treatments and for the understanding of resistance dynamics in pneumococcal infections.

In the analysis by serotypes, Spn14 showed the highest resistance, with 72.3% resistance to penicillin and 66.7% resistance to ceftriaxone, and 20% of the isolates was multiresistant. In addition, 48.6% of the Spn19A strains were resistant to penicillin, 51.1% to ceftriaxone and 82.2% to macrolides, with 65% of the cases exhibiting multiresistance. Spn6C was associated with resistance to macrolides in 59.1% of the patients. Additional details on these resistance profiles can be found in Figure 3 and Supplementary Table S2.

## Discussion

The present study, carried out by the Neumocolombia network in 17 Colombian hospitals, offers a detailed overview of the behavior of the IPD in the context after the implementation of the PCV10 vaccine.

*Streptococcus pneumoniae* is the leading cause of meningitis and pneumonia in children under 5 years of age worldwide and causes 735,000 annual deaths from IPD (1, 2, 13). After the introduction of conjugate vaccines, the incidence of IPD has decreased, but it is crucial to continue with surveillance to monitor clinical behavior, the prevalence of serotypes, and bacterial resistance (1, 4, 20, 27). This study showed that the majority of patients with IPD were under 24 months of age (40.5%), followed by the 24–59 months age group (38.5%). Bacteremic pneumonia is the most common IPD, followed by primary bacteremia and bacterial meningitis, in line with other studies (5, 6, 28–31). A prevalence of 27.6% of complicated pneumonia was observed, consistent with findings that have been reported in other studies, associated with the emergence and prevalence of Spn3 and Spn19A. This has an impact on clinical care given that this high prevalence makes empirical management difficult, leading to the use of broader-spectrum antibiotics, longer treatment, and more need for surgical procedures, increasing the proportion who are admitted to the PICU and the length of hospital stays (5, 31). Primary bacteremia is the second most common IPD, but it is not part of routine surveillance. We believe that epidemiological surveillance should be strengthened when this pathology is included (27, 32).

The use of molecular tests for the diagnosis of IPD has increased the number of confirmed cases, especially bacterial meningitis, complicated pneumonia, and osteoarticular infection; these findings are similar to those found in the world literature (6, 33, 34).

IPD imposes high costs on health systems. In the present study, we found a median hospitalization duration of 12 days (IQR 8–14); 50% of the patients were admitted to the PICU, where they stayed for 9 days (IQR 6–13). The proportion of patients admitted to the PICU was higher than that reported in Spain (19%) (35). The fatality in the study was 12%, which was higher than the 7.5% reported in a previous study that included data between 2008 and 2014 and higher than the 1.3% reported in Spain (35), This difference can be explained by multiple factors, including the higher prevalence of Spn19A and Spn3, which have been associated with a higher likelihood of admission to the PICU and longer hospital stays (5, 15, 16, 22).

The number of *Streptococcus pneumoniae* isolates and the number of serotyped isolates decreased during 2020 and 2021, with an increase in 2022, a behavior similar to that observed in other countries (36, 37). This behavior is related to multiple factors, among which the measures taken during the pandemic (universal use of masks, physical distancing, and increased hand hygiene) decreased the incidence of viral and bacterial infections (37, 38). In the present study, nasopharyngeal carriage was not evaluated, but a study carried out in Israel showed that the incidence of IPD decreased during the pandemic and that the nasopharyngeal carriage of *Streptococcus pneumoniae* did not decrease, highlighting the importance of respiratory viruses in the genesis of the IPD (39). Other factors may also explain the decrease in the number of *Streptococcus pneumoniae* isolates that reached the surveillance system to be serotyped: the decrease in epidemiological surveillance of IPD in some regions as a consequence of the need for systems to rapidly strengthen the surveillance of COVID-19, the shortage of culture media and other reagents, and the difficulties in transporting samples during the pandemic (37). In 2022, the number of isolates increased, as in other countries (40, 41), related to the suspension of the measures implemented during the pandemic, the increase in viral infections, and the presence of immunological debt (40, 41).

The most frequent serotype found during the study period was Spn19A (51.3%), whose proportion increased over time. The same phenomenon has been described in national surveillance data and in other studies conducted in Colombia (4–7, 20, 28–30, 42, 43) and elsewhere around the world after the universal administration of PCV10 (4, 7, 14, 44–46). Serotypes Spn6C and Spn3 have also become more prevalent in all age groups, as has been observed in countries that have administered PCV10 (7, 47). Spn3 has been associated with a longer hospital stay and higher likelihood of admission to the PICU, and Spn6C has been associated with resistance to macrolides. These three serotypes are covered directly or indirectly by PCV13; by the year 2022, it covered 75% of the circulating serotypes in Colombia, which is why the change was made in that same year (15, 16, 48). Due to the high frequency of Spn19A and Spn6C found in the 24- to 59 months-old group in Colombia, the administration of a dose of PCV13 to this age group as a levelling strategy could offer benefits in reducing colonization and infection by these serotypes, similar to that reported in Italy, the United States, and Taiwan (49–53).

In recent years, vaccines with more valences have been developed, and studies have reported that these vaccines can cover more serotypes, depending on the vaccine previously used. It has been estimated that PCV15 could cover more than PCV13 by 4–6% in countries that use PCV10 and 10% in countries that use PCV13. PCV20 has been predicted to increase serotype coverage by 9–15% in the countries that use PCV10, and by 23–26% in the countries that use PCV13 (15, 16). In the present study, no isolates of the additional serotypes included in PCV15 were observed, and isolates of additional serotypes included in PCV20 were observed to represent an additional 3% of PCV13; therefore, at present in Colombia, PCV13 is considered to offer adequate coverage for circulating serotypes (Spn19A, Spn6C, and Spn3). Epidemiological surveillance should continue to be strengthened since it is possible that the emergence of the serotypes included in PCV15 and PCV20 may be observed in the mature phase of PCV13 administration, similar to what has been observed in other countries (3, 54, 55).

Regarding resistance to antibiotics, we found a decreased sensitivity of 41.3% to penicillin and 21.7% to ceftriaxone in meningeal isolates and 28.2% to penicillin and 24.2% to ceftriaxone in nonmeningeal isolates, along with a high proportion of resistance to macrolides, clindamycin, and trimethoprim-sulfamethoxazole. Therefore, these antibiotics should not be used as empirical treatments for pneumococcal infections. These findings are similar to those reported by the National Surveillance System between 2016 and 2021: penicillin resistance in children under 5 years of age was between 33.6 and 70.6% for meningeal isolates and between 30 and 48% for nonmeningeal isolates, the decrease in sensitivity to ceftriaxone for meningeal isolates was between 22 and 25%, and that for nonmeningeal isolates was between 21.6 and 41.6%. Resistance to macrolides ranged between 43.5 and 58.7%, and resistance to trimethoprim-sulfamethoxazole ranged between 43.5 and 57.3% (56). Similar behavior has been observed in other regions of the world (7, 46). The present study found that the proportion of antimicrobial agent resistance was not uniform between the serotypes; some of the antimicrobial agents, such as Spn14, Spn6C and Spn19A, have been associated with high resistance to penicillin, but the latter has been related to the clonal complex CC320, a finding similar to those reported in Brazil, Ecuador, and Belgium (7, 28, 46, 47). This resistance phenomenon could be explained by the presence of circulating clones and by the pressure exerted by the use of antibiotics (7, 28, 57, 58).

Among the strengths of this study are the representativeness of the hospitals that make up the network, the number of cases analyzed, and the clinical information presented. One weaknesses of our methods is the inability to obtain the serotypes of all the isolates, and bias can be detected by obtaining information from medical records. Another potential weakness is the absence of statistical analysis to determine the significance of changes over the observed period, however, after careful deliberation, our team of developers and researchers concluded that a detailed descriptive analysis of the epidemiological behavior and trends would be more relevant and informative for this particular study. Our objective was to provide a comprehensive overview of the pathogen’s distribution over the specified timeframe, thereby offering valuable insights for clinicians and public health decision-makers, especially in the context of vaccine strategy planning. While we acknowledge the value of associative studies, we believed that an in-depth descriptive account would serve as a crucial foundation for future research. This approach ensures that our conclusions are grounded in available the data, avoiding pitfalls of over-interpretation or misinterpretation due to inadequate statistical power.

## Conclusion

IPD continues to be an important cause of morbidity and mortality in children. The number of IPD cases decreased during the pandemic and increased again in 2022. Conjugate pneumococcal vaccines have decreased the incidence of IPD to the extent that they cover the most prevalent serotypes. The phenomenon of serotype exchange can reduce the impact of vaccines. In Colombia, 5 years after the universal administration of PCV10, the incidence of IPD caused by all 10 serotypes decreased, but serotypes Spn19A, Spn3, and Spn6C emerged in all age groups, especially in patients aged 24 to 59 months. Spn19A is associated with multiresistance, Spn3 is associated with a longer hospital stay and a higher likelihood of admission to the PICU, and Spn6C has resistance to macrolides. In Colombia, the change to PCV13 was implemented in July 2022 in the basic 2 + 1 scheme in the first 2 years of life. Based on the data presented, a catch-up or levelling vaccination strategy is recommended for children between 24 and 59 months. In the present study, no isolates of the two additional serotypes included in PCV15 were observed, and the prevalence of the seven additional serotypes of PCV20 was low, so we believe the PCV13 vaccine adequately covers the most prevalent serotypes in Colombia at this time. In the country, epidemiological surveillance should be strengthened to evaluate the impact of PCV13 and to detect the emergence of serotypes not included in this vaccine as early as we can.

## Data availability statement

The original contributions presented in the study are included in the article/Supplementary material, further inquiries can be directed to the corresponding author.

## Ethics statement

The studies involving humans were approved by Fundación Hospital pediátrico de la Misericordia. The studies were conducted in accordance with the local legislation and institutional requirements. Written informed consent for participation in this study was provided by the participants’ legal guardians/next of kin. The research protocol was approved by the Ethics and Research Committee of HOMI, Hospital pediátrico la Misericordia, through Resolution No. 015 CEI 5018, and approved by the ethics and research committees of each participating institution. In hospitals where the research committee requested it (such as Hospital Infantil Universitario de San José y el Hospital Universitario Clínica San Rafael), informed consent was obtained from the parents. In other hospitals, the ethics and research committee waived the need for informed consent, considering it to be a low-risk study.

## Author contributions

GC-M: Conceptualization, Data curation, Formal analysis, Funding acquisition, Investigation, Methodology, Project administration, Resources, Software, Supervision, Validation, Visualization, Writing – original draft, Writing – review & editing. AL: Conceptualization, Formal analysis, Funding acquisition, Investigation, Methodology, Project administration, Resources, Supervision, Validation, Visualization, Writing – original draft, Writing – review & editing. JP-N: Conceptualization, Formal analysis, Investigation, Methodology, Writing – original draft, Writing – review & editing, Resources, Validation. PV-H: Data curation, Formal analysis, Software, Supervision, Writing – original draft, Writing – review & editing. IG: Conceptualization, Investigation, Methodology, Visualization, Writing – review & editing. SB: Conceptualization, Investigation, Methodology, Visualization, Writing – review & editing. MÁ-O: Investigation, Methodology, Visualization, Writing – review & editing. A-CM: Formal analysis, Investigation, Methodology, Visualization, Writing – review & editing. JL-R: Investigation, Visualization, Writing – review & editing. RB: Investigation, Visualization, Writing – review & editing. JR: Investigation, Visualization, Writing – review & editing. FE: Investigation, Visualization, Writing – review & editing. CA-F: Investigation, Visualization, Writing – review & editing. MS: Investigation, Visualization, Writing – review & editing. MT: Investigation, Visualization, Writing – review & editing. EL-M: Investigation, Visualization, Writing – review & editing. PL: Investigation, Visualization, Writing – review & editing. WC: Investigation, Visualization, Writing – review & editing. NR: Investigation, Visualization, Writing – review & editing. AR: Investigation, Methodology, Visualization, Writing – review & editing. AM: Investigation, Visualization, Writing – review & editing. VM: Conceptualization, Data curation, Formal analysis, Funding acquisition, Investigation, Methodology, Project administration, Resources, Software, Supervision, Validation, Visualization, Writing – original draft, Writing – review & editing.
