# Supplementary material for: Serotype distribution, clinical characteristics, and antimicrobial resistance of pediatric invasive pneumococcal disease in Colombia during PCV10 mass vaccination (2017–2022)
Source: Front Med (Lausanne). 2024 May 22;11:1380125. doi: 10.3389/fmed.2024.1380125 (PMC11150640; doi:10.3389/fmed.2024.1380125)
Supplement: Supplementary file 1 [file Data_Sheet_1.docx]

**Table 1s.** Proportion of patients diagnosed through blood and/or fluid cultures (CSF, PF, SF, PeF) and molecular tests.

| Diagnostic | Culture only (Blood culture and/or fluid culture). n(%) | Cultures (blood culture and/or fluid culture) + molecular test | Molecular test only | Total |
| --- | --- | --- | --- | --- |
| Pneumonia:   - Complicated - No complicated | 130 (97)  183 (87.1) | 4 (3)  24 (11.4) | 0  3 (1.4) | 134  210 |
| Bacteriemia | 91(95.7) | 4 (4,3) | 0 | 95 |
| Meningitis | 32 (60.3) | 14 (26,2) | 7 (13.2) | 53 |
| Pneumonia + meningitis | 6 (100) | 0 | 0 | 6 |
| Other diagnoses | 31(96,9) | 0 | 1(3.1) | 32 |
| Total | 473(89.2) | 46 (8.8) | 11(2) | 530 |

*CSF: Cerebrospinal fluid*

*PF: Pleural fluid*

*SF: Synovial fluid*

*PeF: Peritoneal fluid*
