# Supplementary material for: Serotype distribution, clinical characteristics, and antimicrobial resistance of pediatric invasive pneumococcal disease in Colombia during PCV10 mass vaccination (2017–2022)
Source: Front Med (Lausanne). 2024 May 22;11:1380125. doi: 10.3389/fmed.2024.1380125 (PMC11150640; doi:10.3389/fmed.2024.1380125)
Supplement: Supplementary file 2 [file Data_Sheet_2.docx]

**Table 2s**. Distribution of outcomes by serotypes

| Serotype | n | Median hospital stay (IQR) | Average hospital stay | Proportion of patients admitted to the PICU  n(%) | Median PICU stay (IQR) | Average PICU stay | Proportion of decreased sensitivity to penicillin. | Proportion of decreased sensitivity to ceftriaxone | Proportion of decreased sensitivity to macrolides |
| --- | --- | --- | --- | --- | --- | --- | --- | --- | --- |
| 19A | 153 | 12(7-23) | 17 | 81(52.9) | 8(3-12) | 11.6 | 72/148 (48.6%) | 72/141(51.1%) | 116/141 (82.2% |
| 6C | 23 | 13(5-19) | 14.1 | 11(47.8) | 9(5-10) | 9.7 | 1/22 (4.5%) | 0/20 (0%) | 13/22(59.1%) |
| 3 | 20 | 19(12-36) | 25.6 | 15(75) | 12(5-25) | 14.3 | 0/19 (0%) | 0/18(0%) | 3/19(15,8) |
| 6A | 11 | 10(9-12) | 15.5 | 5(45.4) | 5(4-6) | 7 | 0/11 (0%) | 0/9 (0%) | 5/11(45.4%) |
| 14 | 11 | 6(1-7) | 7.3 | 4(36.4) | 7(6-8) | 3.8 | 8/11 (72.3%) | 6/9 (66.7%) | 1/11(9.1%) |
| Other PCV10 serotypes | 7 | 11(10-13) | 10.4 | 3(42.9) | 3(2-6) | 3 | 0/7(0%) | 0/7(0%) | 1/7(14,3%) |
| Other PCV20 non-PCV13 serotypes | 10 | 11(8-14) | 12.6 | 4(40) | 8(3-12) | 8.3 | 1/10(10%) | 0/9(0%) | 1/10(10%) |
| Other non-PCV serotypes | 63 | 14(6-22) | 18.4 | 33(52.4) | 9(4-16) | 15 | 6/63(9.5%) | 3/57(5.3%) | 11/62(17,7%) |
| Total | 298 | 12(7-21) | 16.9 | 156(52.3) | 8(4-14) | 11.9 | 88/291 (30.2%) | 81/270 (30%) | 151/283 (53,3%) |
