# Supplementary material for: Serotype distribution, clinical characteristics, and antimicrobial resistance of pediatric invasive pneumococcal disease in Colombia during PCV10 mass vaccination (2017–2022)
Source: Front Med (Lausanne). 2024 May 22;11:1380125. doi: 10.3389/fmed.2024.1380125 (PMC11150640; doi:10.3389/fmed.2024.1380125)
Supplement: Supplementary file 3 [file Image_1.pdf]

Figure 1s: Proportion of circulating serotypes covered by the vaccines

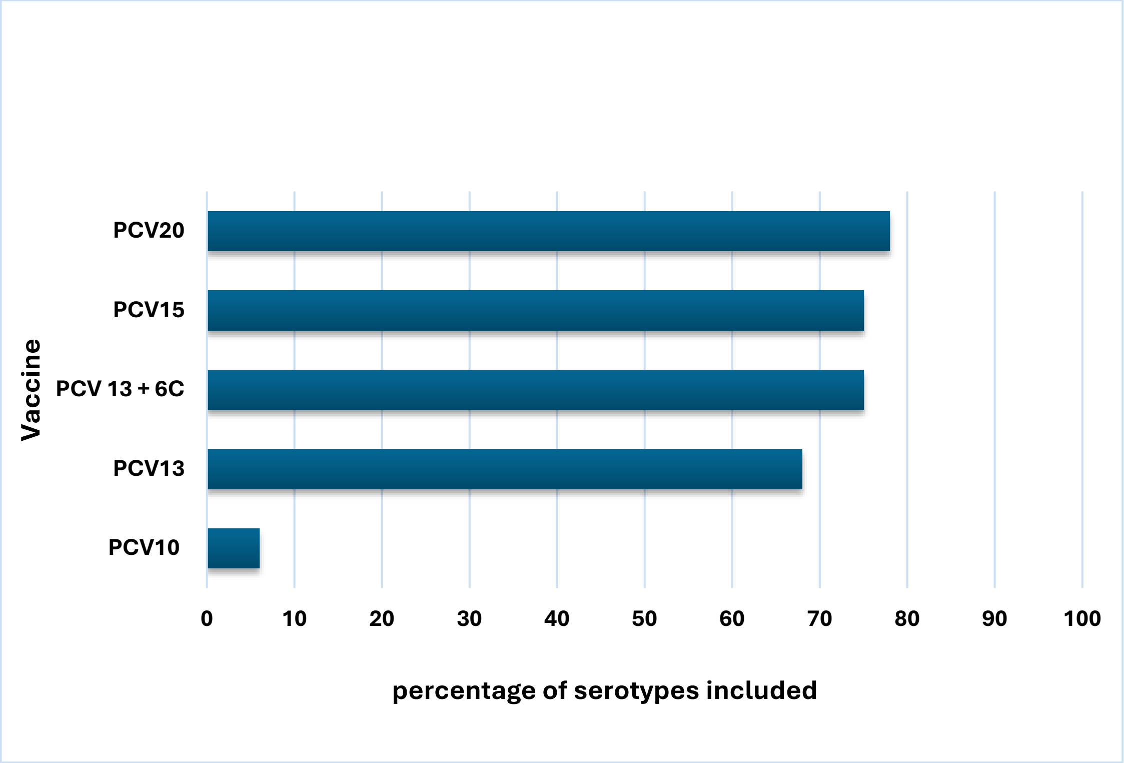

This graph shows the percentage coverage of Streptococcus pneumoniae serotypes by the different conjugate vaccines against pneumococcus. The bars represent the PCV10, PCV13, PCV15 and PCV20 vaccines, and show the percentage of serotypes covered by each. This visualization provides a clear comparison of the effectiveness of different vaccines in covering prevalent serotypes, offering important insight into the potential efficacy of vaccines in preventing pneumococcal diseases.
